# Refractory Thrombocytopenia and Neutropenia: a Diagnostic Challenge

**DOI:** 10.4084/MJHID.2015.018

**Published:** 2015-02-18

**Authors:** Emmanuel Gyan, François Dreyfus, Pierre Fenaux

**Affiliations:** 1 Service d’hematologie et therapie cellulaire, Centre hospitalier universitaire, Tours, France; 2 Team 2 *“Leukemic Niche and Redox metabolism”,* UMR CNRS 7292 GICC, Université François Rabelais, Tours, France; 3 Groupe Francophone des Myélodysplasies, Hôpital Saint Louis, AP-HP, Paris, France; 4 Service d’hematologie, Hotel-Dieu, AP-HP, Paris, France; 5 Service d’hematologie seniors, Hopital Saint Louis, AP-HP and Paris 7 University, Paris, France

## Abstract

The 2008 WHO classification identified refractory cytopenia with unilineage dysplasia (RCUD) as a composite entity encompassing refractory anemia, refractory thrombocytopenia (RT), and refractory neutropenia (RN), characterized by 10% or more dysplastic cells in the bone marrow respective lineage. The diagnosis of RT and RN is complicated by several factors. Diagnosing RT first requires exclusion of familial thrombocytopenia, chronic auto-immune thrombocytopenia, concomitant medications, viral infections, or hypersplenism. Diagnosis of RN should also be made after ruling out differential diagnoses such as ethnic or familial neutropenia, as well as acquired, drug-induced, infection-related or malignancy-related neutropenia. An accurate quantification of dysplasia should be performed in order to distinguish RT or RN from the provisional entity named idiopathic cytopenia of unknown significance (ICUS). Cytogenetic analysis, and possibly in the future somatic mutation analysis (of genes most frequently mutated in MDS), and flow cytometry analysis aberrant antigen expression on myeloid cells may help in this differential diagnosis. Importantly, we and others found that, while isolated neutropenia and thrombocytopenia are not rare in MDS, those patients can generally be classified (according to WHO 2008 classification) as refractory cytopenia with multilineage dysplasia or refractory anemia with excess blasts, while RT and RN (according to WHO 2008) are quite rare. These results suggest in particular that identification of RT and RN as distinct entities could be reconsidered in future WHO classification updates.

## Background: WHO Classification of MDS

Myelodysplastic syndromes (MDS) are marrow stem cell disorders characterized by ineffective hematopoiesis leading to blood cytopenias, a variable proportion of blasts, and a propensity to evolve to acute myeloblastic leukemia (AML). The first classification of MDS was published by the French-American-British group in 1982, individualizing five entities named refractory anemia (RA), refractory anemia with ringed sideroblasts, RA with excess blasts (RAEB), RA with excess blasts in transformation (RAEB-T), and chronic myelomonocytic leukemia (CMML).^1^ This FAB MDS classification, mainly based on the morphologic features of the blood and the bone marrow was refined in 2002^2^ and finally in 2008 by the World Health Organization,^3^ that shifted the RAEB-T category to AML by lowering the threshold of bone marrow blasts for AML diagnosis from 30% to 20%, also excluded CMML from MDS, individualized MDS with isolated deletion of the long arm of chromosome 5 (del 5q), and took into account the number of morphologically dysplastic myeloid lineages. This led to separate, in patients without excess of marrow blasts, those with multilineage dysplasia (refractory cytopenia with multilineage dysplasia or RCMD, with or without ringed sideroblasts) from patients with unilineage dysplasia (refractory cytopenia with unilineage dysplasia or RCUD) (Table 1).

## RCUD as a Distinct Diagnostic Group in the 2008 WHO Classification

RCUD was thus identified as a new MDS group, containing three arbitrarily defined subgroups: refractory anemia (RA), refractory neutropenia (RN) and refractory thrombocytopenia (RT). It is important to consider that these diagnoses are mainly based on the bone marrow finding of a unique dysplastic lineage, contrarily to what their name would intuitively suggest. The characteristics of WHO-defined RCUD are detailed below.

### Common characteristics of RCUD

Marrow findings should be unilineage dysplasia defined as the presence of ≥ 10% dysplastic cells in one myeloid lineage. Less than 5% blasts are observed. The blood should contain < 1% blasts. Cases of unilineage dysplasia with 1% circulating blasts should be classified as MDS-U. If 2–4% circulating blasts are observed, the diagnostic classification is RAEB-1. Even though RARS has unilineage dysplasia, it is recognized as a distinct entity and not included in RCUD. Therefore, RA diagnosis is considered when only erythroid dysplasia is present and if < 15% ringed sideroblasts.

For the diagnosis of MDS, cytopenias are defined as hemoglobin < 10 g/dL, absolute neutrophil count (ANC) < 1.8×10^9^/L, and platelet count < 100×10^9^/L. Importantly, two cytopenias are accepted for the diagnosis of RCUD, provided there is only one dysplastic lineage in the bone marrow. In case of pancytopenia associated with only one dysplasia in the bone marrow, the classification should be MDS-U (Table 1). Also, the cytopenia does not always correspond to the bone marrow dysplastic lineage. In a series of 44 patients with a single cytopenia with unilineage dysplasia described by Verburgh et al, 18 (41%) presented with a cytopenia in a lineage not affected by dysplasia.^4^ This discrepancy creates an ambiguity in the understanding of the RCUD subgroups, theoretically characterized by one ‘refractory cytopenia’ (RA, RN, or RT), since a unique cytopenia in a patient with MDS may be associated in some cases with ≥10% bone marrow dysplasia in another or several lineages. There is thus an ‘unilineage paradox’, where the WHO-defined RCUD can be associated with one or two cytopenias not corresponding with the affected lineage in the bone marrow, whereas MDS with only one cytopenia – which could be identified as ‘isolated thrombocytopenia’ (IT) or ‘isolated neutropenia’ (IN) – are common. This issue will be discussed below.

In refractory anemia (RA), signs of dyserythropoiesis may be observed on blood smears, such as macrocytosis, anisochromasia or dimorphism, with or without anisocytosis and poikilocytosis, which are markers of clonal heterogeneity in a chimeric bone marrow. Neutrophils and platelets are usually normal in number and morphology. However, the presence of moderate neutropenia or thrombocytopenia remains consistent with the diagnosis of RA. Bone marrow cellularity is generally increased, but can be normal or decreased. Dyserythropoiesis is defined as 10% or more dysplastic erythroid precursors. Dysery-thropoiesis is not specific for RCUD compared to other types of MDS. If a dysplasia is present in a second lineage, it should always be < 10%.

In refractory neutropenia (RN), dysgranulopoiesis can be identified in the blood by the presence of nuclear hypolobation and hypogranulation of neutrophils. In the bone marrow, dysplasia in the granulocytic lineage is ≥10%, with no significant dysplasia (<10%) in the erythroid or megakaryocytic lineage.

Refractory Thrombocytopenia (RT) is mainly characterized in the blood by isolated thrombo-cytopenia. A second cytopenia may be associated. In the bone marrow, RT is characterized by ≥10% dysplasia evaluated on at least 30 megakaryocytes. Dysmegakaryopoiesis may include hypolobated megakaryocytes, multinucleated megakaryocytes and micromegakaryocytes. The other cell lineages are not affected, or may display non-significant dysplasia (<10%).

## Differential Diagnosis of RT

Following the exclusion of pseudothrombocytopenia, isolated thrombo-cytopenia of RT should mainly be distinguished from chronic immunologic thrombocytopenic purpura (ITP) and familial thrombocytopenia (Table 2). RT may be overlooked if bone marrow evaluation is not performed. For this reason, the bone marrow examination should be performed in any patient with an isolated confirmed thrombocytopenia above the age of 60 years.^5^ A complete workup for thrombocytopenia should be performed with viral serology, careful medical history with an inquiry about all possible concomitant medications is needed. Cytogenetic studies are of clear interest in this distinction, since 20q deletion has frequently been reported in RT,^6^–^8^ or more rarely other cytogenetic abnormalities such as del(5q).^9^ Furthermore, even in MDS, an autoimmune destruction of platelets can contribute to thrombocytopenia. Platelet lifespan studies (and of their sequestration) by radioisotopic methods can be of interest to analyze the various mechanisms of thrombocytopenia,^10^ and help in therapeutic decision-making.^11^ Anti-platelet autoantibodies have a low sensitivity for the diagnosis of ITP,^12^ and, although they are frequently positive in MDS^13^ but they do not help very much to identify a mixed pathophysiology of thrombocytopenia.^10^ Platelet morphology on blood smears can be helpful for diagnostic orientation. Giant platelets or microthrombocytes can be secondary to hereditary thrombocytopenias of childhood,^14^ or associated infections. Associated morphological abnormalities such as Pelger-Huët bilobed nuclei, or evidence of dysgranulopoiesis may be suggestive of MDS, whereas abnormal hematopoietic cells may orient the diagnosis towards a hematologic malignancy.

## Differential Diagnosis of RN

During workup for neutropenia, sepsis-associated, drug induced, hemodialysis-associated, auto-immune, familial or “ethnic” neutropenia, should be ruled out (Table 3).^15^ Acute or cyclic neutropenias are not consistent with the diagnosis of RN. Post-infectious neutropenia is mostly seen after viral infections such as varicella, rubella, influenza, measles, hepatitis, Epstein-Barr virus or HIV infections, and may sometimes be prolonged. Chronic moderate isolated neutropenia can be secondary to concomitant medications (such as clozapine, chlorpromazine, ticlopidine, or sulfasalazine), auto-immune disorders, and ethnic/familial neutropenias, characterized by an excessive margination of granulocytes.^16^ Autoimmune neutropenia is mainly associated with autoimmune diseases such as lupus erythematosus (LE) or rheumatoid arthritis (Felty’s syndrome),^17^ or large granular lymphocyte leukemia.^18^ Neutropenia associated with other cytopenias may be suggestive of splenomegaly, dietary deficiencies or hematologic malignancies, and should be explored appropriately.

## Getting Appropriate Material for Morphological Diagnosis

The diagnosis of MDS, and particularly RCUD, relies on the availability of high quality bone marrow samples, and on the exclusion of other diseases. Morphological bone marrow examination, with an iron stain and cytogenetic study still represents the cornerstone of MDS diagnosis. In a study comparing bone marrow smears, bone marrow imprints, and bone marrow biopsies, the best accuracy in 86 MDS was achieved with BM smears. Interestingly, for patients with a diagnosis of RCUD, inter-observer accuracy was 100% with BM smears, compared with only 60% with BM sections.^19^

## Distinguishing between RCUD and Borderline Entities

The WHO 2008 classification proposed an entity named idiopathic cytopenia of unknown significance (ICUS), defined as a condition with less than 10% dysplastic cells, fewer than 5% blasts in the bone marrow and no cytogenetic abnormalities.^3^,^20^ These patients most often present with mild cytopenias, and if the morphologist is unaware of the complete medical history, the diagnosis might be reported as “abnormalities not sufficient for the diagnosis of MDS”, when the cytogenetic study is normal. Differential diagnosis of ICUS, like for RCUD, includes autoimmune disorders, drug intake, chronic infections, paroxysmal nocturnal hemoglobinuria, and appropriate explorations need to be carried out.^21^,^22^ ICUS patients should be followed to document or exclude hematological evolution to an authentic MDS, most importantly by repetition of the BM examination with cytogenetic studies if the cytopenia worsens or if a second cytopenia develops. One should also bear in mind that dysplastic changes can be seen in up to 9,5% of the erythroid or granulocytic bone marrow cells in elderly persons and in smokers.^23^

Another borderline entity is idiopathic dysplasia of unknown significance (IDUS). This is a rare condition characterized by no or only mild cytopenias (hemoglobin ≥11 g/dL, neutrophils ≥1500/mm3, and platelets ≥100000/mm3, associated with > 10% dysplasia in one lineage.^24^ Most patients are asymptomatic young patients referred to the hematology departments because of macrocytosis or detection of Pseudo-Pelger-Huët abnormalities. As for ICUS, these patients should have regular follow-up and repeated diagnostic investigations in case of hematologic evolution, likely to detect overt MDS. To harmonize the identification of the minimal changes sufficient for MDS diagnosis, a recent collaborative work has set up a list of morphological findings with a high sensitivity/specificity, a high reproducibility and a high prognostic value of a morphology-based score.^25^

The role of cytogenetic analysis is important in the identification of RCUD, since cytogenetic abnormalities will support the diagnosis of MDS as opposed to ICUS.^21^ The most common cytogenetic abnormality in RCUD is del(20q). In a cytogenetic and mutational study of 305 MDS with del(20q) whose samples were referred to the MLL Munich Leukemia laboratory, the most represented diagnostic category was RCUD (133 patients, 43.6%), among which 80.5% had del(20q) as sole abnormality.^26^ High-throughput sequencing can also help in the diagnosis of MDS in difficult cases by detecting mutations frequently associated with MDS, including TET-2, ASXL1, SF3B1, SRSF2, RUNX1 and DNMT3A.^27^,^28^ On the other hand isolated mutations of TET2, ASXL1 or DNMT3a can be found in elderly apparently healthy persons.^29^

## RT and RN are Rare

Apart from RA, the other RCUD (RT and RN) appear to be rare. In a cytomorphologic study of 3156 MDS patients from the Düsseldorf MDS registry, the diagnosis of RCUD was made in 218 (7%). When the Düsseldorf group revaluated, by WHO 2008 diagnostic criteria, 193 RA according to WHO 2001, the following diagnoses were found: 37 RCUD (19%), 6 MDS-U (3%), 111 RCMD (58%), and 39 5q- syndromes (20%), but a higher proportion of RCUD (45%) was found in the Japanese registry.^30^ To assess the RCUD and MDS-U categories in 196 patients with less than 5% marrow blasts, Maassen et al. found 28% RA, 6% RT, 13% RN, 20% patients with no cytopenia, and 34% patients with bicytopenia.^31^ Another retrospective study on 293 MDS in a single institution identified 5 RN (1.7%) and 6 RT (2.0%) only.^32^ Furthermore, in a study combining 228 MDS patients from the Italian, Düsseldorf and GFM registries presenting with isolated neutropenia (IT) (< 1.5 × 10^9^/L) or isolated thrombocytopenia (IT) (< 100 × 10^9^/L) and no anemia, we found only 3 (1%) RT and no RN (Gyan et al., submitted). The most frequent diagnosis found in patients with IT or IN was RCMD (32 %) and RAEB-1 (18 %), which occurred at similar frequency in both types. Furthermore, during evolution, RT or RN patients often develop additional cytopenias,^33^ which is consistent with the hypothesis that RT and RN are early presentations of refractory cytopenias with multilineage dysplasia. This observation further suggests that real WHO-defined RT and RN are very rare – if they even exist – whereas MDS patients with only one cytopenia most often show dysplasia in multiple lineages.

Another important issue adds to the difficulty of identifying RT and RN. Following publication of the WHO 2008 classification, a study evaluating the inter-observer variability in MDS diagnosis found a discrepancy rate of 27%, mostly in the categories with unilineage dysplasia.^34^ This was recently confirmed by a study of 50 cases of unilineage dysplasia where an agreement of only 21% was present between observers. Additionally, the threshold of 2% blasts for the revised IPSS calculation was subject to a 30% discordance rate.^35^ The diagnosis of RT or RN thus remains difficult and does not to date reflect an international and reliable consensus on diagnostic criteria. The fact that these extremely rare entities are at the frontiers of RCMD and ICUS/IDUS may be a likely explanation.

## Prognosis of RT and RN

RCUD is associated with a more favorable outcome than RCMD.^4^,^36^ In a comparative study between the Düsseldorf and the Japanese MDS registries, median overall survival of RCUD and RCMD was 202 months vs. 109 months in the Japanese cohort, respectively, and 142 months vs. 36 months in the German cohort, respectively, with statistical significance.^30^ It is important to try to distinguish RCUD patients with a high and low risk of evolution to RAEB or AML. In a series of 126 patients with RCUD, RT diagnosis was associated with shorter OS (median 15.9 months) then RA (median 48.2 months) and RN (median 35.9 months, p<0.001).^33^ In another study, the number of RT and RN was too low to identify a statistically different outcome, but median survival was 32.5 months and 72 months for RT and RN, respectively.^32^ In a bone marrow flow cytometry analysis of patients with RCUD, Oka et al. described a lower content of CD19+ or CD10+ lymphoid cells in the marrow blast region (CD45^int^/side scatter^low^) of patients in whom circulating blasts appeared during follow-up, compared to patients who did not experience disease evolution to higher risk MDS or AML.^37^

In a study evaluating the prognostic value of multilineage dysplasia, Verburgh et al. found a favorable impact of unilineage dysplasia and of a single dysplasia.^4^ ANC < 500/mm^3^ has been described as an adverse prognostic factor in Low/Int-1 risk MDS by two independent teams, with a shorter leukemia-free survival but surprisingly, no increase in infection-related deaths.^38^,^39^ Beyond the number or cytopenias, the depth of neutropenia and thrombocytopenia have been incorporated as prognostic factors into the revised IPSS prognostic score.^40^

## Diagnostic Tools for the Diagnosis of RT and RN

Flow cytometry (FC) is able to identify aberrant expression patterns of lineage antigens in the erythroid, granulo-monocytic and lymphoid lineages, and a collaborative effort has proposed guidelines for the FC recognition of dysplasia.^41^ Since RCUD displays a variable level of dysplastic cells in one lineage only, FC may be a valuable tool for the identification of MDS FC signatures. Moreover, a FC score may help to distinguish MDS from other nonmalignant reactive or secondary cytopenias,^42^,^43^ and support the diagnosis of IDUS,^24^ which may represent a pre-phase of MDS. The Ogata score, based on a 4-color analysis of 13 antigens, has shown a sensitivity of 70% and a specificity of 92% in the whole MDS group.^43^ For RCUD, the sensitivity was 62%, and a specificity reaching 97% in distinguishing MDS from immune cytopenias.^43^ Additionally, a FC score is likely to bring prognostic information in MDS even when the blast count is below 5%, with a high correlation with transfusion dependency, cytogenetics, and the IPSS score.^44^ In addition, a higher number of aberrantly expressed antigens detected by FC has been associated with worse survival.^45^ Altogether, the available data support the use of FC as a diagnostic tool to increase the accuracy of RCUD diagnosis, as well as for the diagnosis of differential conditions, such as PNH.

Identification of recurrent mutations with deep sequencing, such as TET-2, ASXL1, TP53, RAS, SF3B1, SRSF2, RUNX1 and others^46^ may help to delineate RN and RT from other non-MDS conditions. However, as said above, mutational analysis as a tool for RT or RN diagnosis may be hampered by the fact that mutations of TET2, DNMT3a and ASXL1 can be seen individually in elderly healthy persons.^29^

## Conclusions

The challenge of RT and RN resides in the paucity of diagnostic criteria, the possible overlap with non-MDS disorders, and in the rarity of true cases of these subgroups of RCUD. Furthermore, isolated refractory cytopenias are frequent in other MDS categories. The workup of such patients should include a complete screening for differential diagnosis, cytogenetic analysis, an expert review of the bone marrow smears, and the help of emerging diagnostic tools such as flow cytometry and molecular biology. The clinical relevance of their distinction from RA or RCMD could be reconsidered in a future revision of the WHO classification of MDS.

## Figures and Tables

**Table 1:** WHO 2008 classification of MDS^3^

	Blood findings	Bone marrow findings
RCUD	single or bi-cytopenia	dysplasia in ≥10% of 1 cell line < 5% blasts
RA		dysplasia in ≥ 10% of the erythroid cell line < 5% blasts
RN		dysplasia in ≥ 10% of the granulocytic cell line < 5% blasts
RT		dysplasia in ≥ 10% of the megakaryocytic cell line < 5% blasts
RARS	anemia, no blasts	≥ 15% of erythroid precursors with ring sideroblasts, erythroid dysplasia only < 5% blasts
RCMD	cytopenia(s), < 1 × 10^9^/L monocytes	dysplasia in ≥ 10% of cells in ≥ 2 hematopoietic lineages ±15% ring sideroblasts, < 5% blasts
RAEB-1	cytopenia(s) ≥ 2–4% blasts < 1 × 10^9^/L monocytes	unilineage or multilineage dysplasia no Auer rods 5–9% blasts
RAEB-2	5–19% blasts < 1 × 10^9^/L monocytes	unilineage or multilineage dysplasia or Auer rods or 10–19% blasts
5q-	anemia, platelet levels normal or increased	unilineage erythroid dysplasia, isolated del(5q) < 5% blasts
MDS-U	cytopenias	unilineage dysplasia with pancytopenia or no dysplasia but characteristic MDS cytogenetics, < 5% blasts

**Table 2:** Differential diagnosis of RT

Pseudothrombocytopenia
Congenital
Familial thrombocytopenia
Wiskott-Aldrich syndrome
Gray platelet syndrome
Bernard-Soulier syndrome
X-linked thrombocytopenia
Acquired
Autoimmune
Immunologic Thrombocytopenic Purpura
Aplastic anemia
Septicemia
Medications
Heparin-induced thrombocytopenia
Drug-induced immune thrombocytopenia
Disseminated intravascular coagulation
Splenomegaly
Portal hypertension, cirrhosis
Gaucher’s disease
Myelofibrosis with myeloid metaplasia
Viral infections
HIV
HCV
Microangiopathy
TTP
Hemolytic uremic syndrome
Malignancy
MDS
Leukemia
Lymphoma
CLL

Abbreviations: CLL, chronic lymphocytic leukemia. HCV, Hepatitis C virus. HIV, human immunodeficiency virus. MDS, myelodysplastic syndrome. TTP: thrombotic thrombocytemic purpura.

**Table 3:** Differential diagnosis of RN

Congenital
Constitutional neutropenia
Ethnic neutropenia
Benign familial neutropenia
Cyclic neutropenia
Acquired
Autoimmune
LE
Felty syndrome
Drug-induced
Agranulocytosis
Mild neutropenia
Late neutropenia
Infection-associated
Active infection
Viral infections
Severe sepsis
Post-infectious
Hemodialysis
Splenomegaly
Malignancy
Acute leukemia
MDS
LGL leukemia
Myeloma, lymphoma
Myelophthisic processes
Dietary
B12, folate deficiency
Copper deficiency
Malnutrition

Abbreviations: LE, lupus erythematosus. MDS, myelodysplastic syndrome. LGL, large granular lymphocyte.

## References

[1] Bennett JM, Catovsky D, Daniel MT (1982). Proposals for the classification of the myelodysplastic syndromes. Br J Haematol.

[2] Jaffe ES (2002). Pathology and Genetics: Tumours of Haematopoietic and Lymphoid Tissues.

[3] Swerdlow SH, Campo E, Harris NL (2008). Who Classification of Tumors of Haematopoietic and Lymphoid Tissues.

[4] Verburgh E, Achten R, Louw VJ (2007). A new disease categorization of low-grade myelodysplastic syndromes based on the expression of cytopenia and dysplasia in one versus more than one lineage improves on the WHO classification. Leukemia.

[5] Provan D, Stasi R, Newland AC (2009). International consensus report on the investigation and management of primary immune thrombocytopenia. Blood.

[6] Padhi S, Varghese R, Phansalkar M, Sarangi R (2013). Isolated deletion of the long arm of chromosome 20 [del(20q12)] in myelodysplastic syndrome: a case report and literature review. Singapore Med J.

[7] Sashida G, Takaku T-I, Shoji N (2003). Clinico-hematologic features of myelodysplastic syndrome presenting as isolated thrombocytopenia: an entity with a relatively favorable prognosis. Leuk Lymphoma.

[8] Haase D, Fonatsch C, Freund M (1995). Cytogenetic findings in 179 patients with myelodysplastic syndromes. Ann Hematol.

[9] Chang J, Park C-J, Seo E-J (2014). A case of refractory thrombocytopenia with 5q deletion: myelodysplastic syndrome mimicking idiopathic thrombocytopenic purpura. Ann Lab Med.

[10] Hebbar M, Kaplan C, Caulier MT (1996). Low incidence of specific anti-platelet antibodies detected by the MAIPA assay in the serum of thrombocytopenic MDS patients and lack of correlation between platelet autoantibodies, platelet lifespan and response to danazol therapy. Br J Haematol.

[11] Sarpatwari A, Provan D, Erqou S (2010). Autologous 111 In-labelled platelet sequestration studies in patients with primary immune thrombocytopenia (ITP) prior to splenectomy: a report from the United Kingdom ITP Registry. Br J Haematol.

[12] Chan H, Moore JC, Finch CN (2003). The IgG subclasses of platelet-associated autoantibodies directed against platelet glycoproteins IIb/IIIa in patients with idiopathic thrombocytopenic purpura. Br J Haematol.

[13] Chabannon C, Molina L, Pégourié-Bandelier B (1994). A Review of 76 Patients with Myelodysplastic Syndromes Treated with Danazol. Cancer.

[14] Patel PD, Samanich JM, Mitchell WB, Manwani D (2011). A unique presentation of Wiskott-Aldrich syndrome in relation to platelet size. Pediatr Blood Cancer.

[15] Gibson C, Berliner N (2014). How we evaluate and treat neutropenia in adults. Blood.

[16] Bishop CR, Rothstein G, Ashenbrucker HE, Athens JW (1971). Leukokinetic studies. XIV. Blood neutrophil kinetics in chronic, steady-state neutropenia. J Clin Invest.

[17] Campion G, Maddison PJ, Goulding N (1990). The Felty syndrome: a case-matched study of clinical manifestations and outcome, serologic features, and immunogenetic associations. Medicine (Baltimore).

[18] Saway PA, Prasthofer EF, Barton JC (1989). Prevalence of granular lymphocyte proliferation in patients with rheumatoid arthritis and neutropenia. Am J Med.

[19] Gong X, Lu X, Wu X (2012). Role of bone marrow imprints in haematological diagnosis: a detailed study of 3781 cases. Cytopathology.

[20] Wimazal F, Fonatsch C, Thalhammer R (2007). Idiopathic cytopenia of undetermined significance (ICUS) versus low risk MDS: the diagnostic interface. Leuk Res.

[21] Giagounidis A, Haase D (2013). Morphology, cytogenetics and classification of MDS. Best Pract Res Clin Haematol.

[22] Valent P, Horny H, Bennett JM (2007). Definitions and standards in the diagnosis and treatment of the myelodysplastic syndromes: Consensus statements and report from a working conference. Leuk Res.

[23] Fernández-Ferrero S, Ramos F (2001). Dyshaemopoietic bone marrow features in healthy subjects are related to age. Leuk Res.

[24] Valent P, Horny H-P (2009). Minimal diagnostic criteria for myelodysplastic syndromes and separation from ICUS and IDUS: update and open questions. Eur J Clin Invest.

[25] Della Porta MG, Travaglino E, Boveri E (2015). Minimal morphological criteria for defining bone marrow dysplasia: a basis for clinical implementation of WHO classification of myelodysplastic syndromes. Leukemia.

[26] Bacher U, Haferlach T, Schnittger S (2014). Investigation of 305 patients with myelodysplastic syndromes and 20q deletion for associated cytogenetic and molecular genetic lesions and their prognostic impact. Br J Haematol.

[27] Bejar R, Stevenson K, Abdel-Wahab O (2011). Clinical effect of point mutations in myelodysplastic syndromes. N Engl J Med.

[28] Bejar R, Stevenson KE, Caughey Ba (2012). Validation of a prognostic model and the impact of mutations in patients with lower-risk myelodysplastic syndromes. J Clin Oncol.

[29] Jaiswal S, Fontanillas P, Flannick J (2014). Age-Related Clonal Hematopoiesis Associated with Adverse Outcomes. N Engl J Med.

[30] Matsuda A, Germing U, Jinnai I (2010). Differences in the distribution of subtypes according to the WHO classification 2008 between Japanese and German patients with refractory anemia according to the FAB classification in myelodysplastic syndromes. Leuk Res.

[31] Maassen A, Strupp C, Giagounidis A (2013). Validation and proposals for a refinement of the WHO 2008 classification of myelodysplastic syndromes without excess of blasts. Leuk Res.

[32] Marinier DE, Mesa H, Rawal A, Gupta P (2010). Refractory cytopenias with unilineage dysplasia: a retrospective analysis of refractory neutropenia and refractory thrombocytopenia. Leuk Lymphoma.

[33] Breccia M, Latagliata R, Cannella L (2010). Refractory cytopenia with unilineage dysplasia: analysis of prognostic factors and survival in 126 patients. Leuk Lymphoma.

[34] Font P, Loscertales J, Benavente C (2013). Inter-observer variance with the diagnosis of myelodysplastic syndromes (MDS) following the 2008 WHO classification. Ann Hematol.

[35] Font P, Loscertales J, Soto C (2014). Interobserver variance in myelodysplastic syndromes with less than 5 % bone marrow blasts: unilineage vs. multilineage dysplasia and reproducibility of the threshold of 2 % blasts. Ann Hematol.

[36] Cazzola M (2011). Risk assessment in myelodysplastic syndromes and myelodysplastic/myeloproliferative neoplasms. Haematologica.

[37] Oka S, Muroi K, Fujiwara S (2012). Prediction of Progression from Refractory Cytopenia with Unilineage Dysplasia by Analysis of Bone Marrow Blast Cell Composition. J Clin Exp Hematop.

[38] Cordoba I, Gonzalez-Porras JR, Such E (2012). The degree of neutropenia has a prognostic impact in low risk myelodysplastic syndrome. Leuk Res.

[39] Breccia M, Loglisci G, Salaroli A (2012). Neutropenia at baseline could indicate poor prognosis in low/intermediate risk myelodysplastic syndrome patients. Leuk Res.

[40] Greenberg PL, Tuechler H, Schanz J (2012). Revised international prognostic scoring system for myelodysplastic syndromes. Blood.

[41] Westers TM, Ireland R, Kern W (2012). Standardization of flow cytometry in myelodysplastic syndromes: a report from an international consortium and the European LeukemiaNet Working Group. Leukemia.

[42] Ogata K, Kishikawa Y, Satoh C (2006). Diagnostic application of flow cytometric characteristics of CD34+ cells in low-grade myelodysplastic syndromes. Blood.

[43] Della Porta MG, Picone C, Pascutto C (2012). Multicenter validation of a reproducible flow cytometric score for the diagnosis of low-grade myelodysplastic syndromes: results of a European LeukemiaNET study. Haematologica.

[44] Van de Loosdrecht AA, Westers TM, Westra AH (2008). Identification of distinct prognostic subgroups in low- and intermediate-1-risk myelodysplastic syndromes by flow cytometry. Blood.

[45] Kern W, Haferlach C, Schnittger S, Haferlach T (2010). Clinical utility of multiparameter flow cytometry in the diagnosis of 1013 patients with suspected myelodysplastic syndrome: correlation to cytomorphology, cytogenetics, and clinical data. Cancer.

[46] Kohlmann A, Bacher U, Schnittger S, Haferlach T (2014). Perspective on how to approach molecular diagnostics in acute myeloid leukemia and myelodysplastic syndromes in the era of next-generation sequencing. Leuk Lymphoma.

